# Research progress on the current status of respiratory pathogen infections and their detection methods

**DOI:** 10.3389/fmicb.2026.1712752

**Published:** 2026-01-23

**Authors:** Fuhong Zhu, Mei Peng, An’ning Chen, Qian-Ying Zhu

**Affiliations:** Department of Laboratory Medicine, The Eighth Affiliated Hospital, Sun Yat-sen University, Shenzhen, China

**Keywords:** atypical pathogen, bacteria, detection method, pathogen, respiratory tract infections, virus

## Abstract

Respiratory tract infections (RTIs) are among the most prevalent diseases in human society and pose a major global health threat, affecting millions annually. A wide range of pathogens, primarily viruses and bacteria, cause RTIs. These infections often present with similar symptoms, which limits effective clinical treatment. Extensive research has addressed RTIs, with ongoing discussion regarding their current status and advancements in detection technologies. Novel laboratory methods that offer rapid, sensitive, and specific results now supplement traditional diagnostic approaches. In this review, we summarize the infection characteristics and detection methods of common respiratory pathogens, evaluate the effectiveness and limitations of current detection methods, and aim to promote advancements in laboratory diagnosis and explore the potential of emerging technologies in this field.

## 1 Introduction

Respiratory tract infections impose substantial health risks and economic burdens on both individuals and society. According to the World Health Organization (WHO), there are annually 3–5 million severe RTI cases and 290,000–650,000 deaths globally (World Health Organization [WHO], 2025). These include upper respiratory tract infections (URTI) and lower respiratory tract infections (LRTI). The GBD study reported that in 2021 (GBD 2021 Upper Respiratory Infections Otitis Media Collaborators, 2024), there were 12.8 billion new cases of URTI worldwide, with an incidence rate of 162,484.8 per 100,000 population. The incidence rate was highest in children under 2 years old, and the number of cases was largest in children aged 5–9. The mortality rate of URTI was 0.2 per 100,000 in 2021. Although the disease burden of LRTI has declined over the past three decades, LRTI caused about 2.5 million deaths in 2021, with a mortality rate of 31.2 per 100,000. LRTI has become the world’s deadliest infectious disease after COVID-19 and is the fourth leading cause of global death (GBD 2021 Causes of Death Collaborators, 2024; World Health Organization [WHO], 2024). This study aims to clarify the typical pathogen spectrum and infection characteristics, summarize the current commonly used detection methods, and discuss the research progress of novel detection methods.

Research confirms that viruses are the primary cause of acute respiratory infections in China, with Influenza Virus (IFV) and Respiratory Syncytial Virus (RSV) being the most common viral pathogens (Zhang et al., 2022). Human Rhinovirus (HRV) and Human Metapneumovirus (HMPV) are also important pathogens (Du et al., 2022). *Streptococcus pneumoniae* (*S. pneumoniae*) and *Mycoplasma pneumoniae* (Mp) account for a significant proportion of bacterial infections (Wang et al., 2022). In Western countries, IFV and RSV are still prevalent, but the incidence and mortality rates of *S. pneumoniae* have been significantly reduced due to widespread vaccination (Kang et al., 2023). The Global Burden of Disease (GBD) study shows that high-income countries, characterized by comprehensive vaccination strategies, have effectively reduced the burden of preventable bacterial infections (Birck et al., 2021). In contrast, China is in a transitional phase of vaccination and has notably lower coverage of the pneumococcal conjugate vaccine (PCV). As a result, the burden of bacterial infections such as pneumococcal disease in China has not been significantly reduced (Yan et al., 2023).

Early childhood LRTI is significantly associated with reduced lung function and increased asthma risk during school age in China (Van Meel et al., 2018). Similar findings have been reported in Western countries, although asthma management strategies may be more robust (Van Meel et al., 2018). The mortality rate of LRTI among Chinese adults may be associated with cold environments, air pollution, and insufficient medical resources (Chen S. et al., 2025), whereas in Western countries, LRTI is more commonly observed in immunocompromised patients or as nosocomial infections (Reyes et al., 2025). Additionally, antibiotic overuse may be more pronounced in China, whereas Western countries increasingly rely on procalcitonin-guided antibiotic management (Tsalik et al., 2023). In temperate regions, including China and some Western countries, RSV and influenza virus epidemics typically peak during winter with distinct seasonality (Robertson et al., 2021; Bolt Botnen et al., 2025). In tropical regions, seasonal patterns may be less pronounced or associated with rainy seasons (Neumann and Kawaoka, 2022). The epidemiology of LRTI in China and Western countries shares similarities in pathogen spectrum, high-risk populations, and climate associations, but differs in disease burden, antibiotic stewardship, healthcare resource allocation, and prevention strategies. The LRTI burden of China may be more driven by environmental and socioeconomic factors, whereas Western countries tend to focus more on healthcare-associated infections and individualized treatment.

## 2 The pathogen spectrum and infection characteristics of RTIs

### 2.1 The pathogen spectrum of RTIs

In the past decade, numerous studies have demonstrated significant variations in the prevalence and pathogen spectrum of respiratory tract pathogens across different countries, regions, populations, years, and seasons. This review selected pathogens mainly by their global and regional epidemiological significance, disease burden, and public health impact. Common pathogenic microorganisms encompass viruses, bacteria, mycoplasma, and chlamydia. A 10-year study in China shows that (Li Z. J. et al., 2021), acute URTI is predominantly caused by viruses, with bacteria accounting for a small proportion. The primary pathogens include IFV (32.8%), HRV (16%), RSV (14.9%), Human Parainfluenza Virus (HPIV, 12.2%), Human Adenovirus (10.5%), Human Coronavirus (HCoV, 6%), Human Bocavirus (3.8%), and HMPV (3.8%). Among the aforementioned, RSV, IFV, HPIV, and HMPV are major pathogens for acute LRTI (Shi et al., 2015). Pneumonia and bronchiolitis due to acute LRTI are the leading causes of global childhood hospitalizations. In 2019, severe acute respiratory infections killed millions of young children, 99% of whom were in developing countries (McAllister et al., 2019; GBD 2019 LRI Collaborators, 2022). Besides viruses, a significant proportion of pathogens are bacteria. The common bacterial pathogens mainly include *S. pneumoniae* (29.9%), Mp (18.6%), *Haemophilus influenzae* (*H. influenzae*, 15.8%), *Klebsiella pneumoniae* (*K. pneumoniae*, 12.5%), *Pseudomonas aeruginosa* (*P. aeruginosa*, 11.4%), *Staphylococcus aureus* (8.9%), *Chlamydophila pneumoniae* (1.6%), and *Legionella pneumophila* (0.9%). Common respiratory fungi include *Aspergillus, Candida*, Mucorales, and *Pneumocystis* (Heung et al., 2023). We have summarized susceptible populations, clinical features, epidemiology, and preferred detection method of common respiratory pathogens in Table 1.

**TABLE 1:** Susceptible populations, clinical features, epidemiology, and preferred detection method of common respiratory pathogens.

Pathogen	Susceptible populations	Clinical features	Epidemiology	Preferred detection method	References
Influenza virus	Children under age 5, adults aged 65 and older, pregnant women, and individuals with compromised immunity	Fever, fatigue, cough, headache, body aches, and other systemic symptoms are prominent.	In temperate regions, this typically occurs during winter and spring, from November to March; in tropical and subtropical regions, it peaks during the rainy season	Multiplex qRT-PCR	Kakoullis et al., 2023; Wang et al., 2023e; Zhou et al., 2017
Respiratory syncytial virus	Infants and toddlers under 6 months old, the elderly, and individuals with compromised immunity	Acute URTI: rhinitis, sore throat, cough, laryngitis; Acute LRTI: bronchitis, pneumonia	Primarily during winter and early spring in temperate regions, with less pronounced incidence in tropical areas, where infection rates are relatively higher during the rainy season	qRT-PCR/LFIA*	Fretzayas and Moustaki, 2017; Szymański et al., 2025; Li Q. et al., 2024; Peters et al., 2017
Human rhinovirus	Infants and young children, the elderly, and immunocompromised individuals	Acute upper respiratory tract infection: nasal congestion, runny nose, sore throat, reduced sense of smell, and mild fever; LRTI is rare	Prevalent throughout the year, but the peak infection periods are in autumn and spring	qRT-PCR	Giardina et al., 2022; Morelli et al., 2025; Lee et al., 2021
Human parainfluenza virus	Children under age 5, the elderly, and individuals with compromised immunity	URTI: flu-like symptoms; LRTI: bronchitis, pneumonia; In rare cases, may lead to otitis media or myocarditis	Primarily transmitted in temperate regions, HPIV-1 and HPIV-3 are most prevalent during the summer and autumn seasons	Multiplex qRT-PCR	Wang et al., 2023b; Li Y. et al., 2023; Lee et al., 2021
Coronavirus	The general population is susceptible. The elderly, children, and immunocompromised individuals are at higher risk of severe respiratory symptoms.	Four seasonal coronaviruses cause mild to moderate URTI; Three highly pathogenic coronaviruses cause a wide range of symptoms, from URTI to severe acute respiratory syndrome	Temperature-sensitive, with infections primarily occurring during winter and early spring; the transmission of some coronaviruses shows no distinct seasonal patterns	qRT-PCR/LFIA*	Shah et al., 2022; Zhu et al., 2020; Rayan, 2021; Bolinger et al., 2024; Lee et al., 2021
Human Metapneumovirus	Infants and young children, the elderly, and immunocompromised individuals	Acute URTI: nasal congestion, cough, fever; Acute LRTI: bronchitis, pneumonia	It can be infected throughout the year, but it is more common in late winter and early spring	qRT-PCR	Acharya and Byrareddy, 2025; Miyakawa et al., 2025; Lim et al., 2020; Lee et al., 2021
*Streptococcus pneumoniae*	Children under age 5, seniors aged 65 and above, and individuals with compromised immunity are high-risk groups for IPD	Otitis media, bronchitis, fever, cough, chest pain	High prevalence in tropical regions may bring pathogens into non-endemic areas by travelers. Winter and early spring are associated with co-infection of respiratory viruses	qPCR/Culture^#^	Narciso et al., 2025; Berry et al., 2020; Vander Elst et al., 2025; Miller et al., 2011; Oyewole et al., 2022
*Mycoplasma pneumoniae*	Children, teenagers, young adults, and those with allergic constitutions	Fever, cough, sore throat, shortness of breath, and moist rales on auscultation of the lungs	Asia (China and Japan have high drug resistance rates), Europe; Summer and early autumn are the peak seasons	qPCR	Pereyre et al., 2016; Cai et al., 2024; Huber et al., 2020
*Haemophilus influenzae*	Children, people with weakened immune systems, and people from specific regions	Otitis media, pneumonia and exacerbation of chronic respiratory diseases	Europe, Asia; High incidence in winter	Culture with AST/qPCR	Tsang et al., 2017; Gozum et al., 2020; Akrami et al., 2023
*Klebsiella pneumoniae*	Newborns, children, the elderly, people with weakened immune systems, hospitalized patients, and patients with chronic respiratory diseases	High fever, cough, purulent sputum, chest pain, lower respiratory tract infection, and empyema; Highly virulent strains can cause invasive infections such as lung abscess	The CRKP is globally distributed and prevalent in Asia, Southern Europe, South America and other regions. No obvious seasonality	Culture with AST/qPCR	Sianturi et al., 2025; Li L. et al., 2023; Liu et al., 2024
*Pseudomonas aeruginosa*	The elderly, those with weakened immune systems, people who have been using ventilators for a long time, and patients with chronic respiratory diseases	Fever, cough, expectoration, breathing difficulties, bronchiectasis, atelectasis, pneumonia	It is globally prevalent, prevalent within hospitals, and has no obvious seasonality	Culture with AST	Tamma et al., 2022; Dong et al., 2025; Bobenchik et al., 2017
*Aspergillus*	Individuals with compromised or deficient immune systems, allergies, or a history of lung disease	It can present as allergic, chronic pulmonary, or invasive forms, such as invasive pulmonary aspergillosis and allergic bronchopulmonary aspergillosis	Aspergillosis occurs worldwide. Incidence rates depend on region, healthcare standards, and diagnostics. Invasive pulmonary aspergillosis is most common, and in China, pulmonary aspergillosis is the predominant clinical form	GM Test/mNGS/qPCR	Moldoveanu et al., 2021; Chirumbolo et al., 2024; Erman-Daloglu et al., 2020
*Candida*	Individuals with immunosuppression or severe underlying medical conditions	Respiratory tract colonization is common. Candidal pneumonia is rare and often hidden by the underlying disease or concurrent infections. Patients with primary candidal pneumonia typically have extreme immunosuppression and face a high mortality rate	*Candida* is globally widespread and commonly colonizes the respiratory tract, serving as one of the major pathogens responsible for hospital-acquired infections	Culture/mNGS	Azoulay et al., 2006; Ahmed et al., 2025; Papon et al., 2020
Mucorales	Individuals with compromised immune function or underlying medical conditions	A severe but relatively rare invasive fungal infection characterized by a strong tendency to invade blood vessels, progressing rapidly and potentially leading to respiratory failure	The pathogen is widespread and considered rare, but its incidence rises in high-risk groups. The pulmonary form is most common globally. In Asia, diabetes is a main risk factor, and the naso-orbito-cerebral form often occurs	qPCR/mNGS/microscopy and culture	Panda et al., 2024; Skiada et al., 2024; Ganesan et al., 2022; Tang et al., 2025
*Pneumocystis jirovecii*	Commonly found in individuals with HIV infection and those with compromised immune function	People with healthy immune systems usually show no symptoms or only mild ones, but immunocompromised people can develop severe, life-threatening PCP	PCP occurs worldwide and mainly affects people with latent infections. It also spreads between people, especially in hospitals where immunocompromised patients gather	qPCR/microscopy and culture	Ames et al., 2023; Wills et al., 2021; Senecal et al., 2022

*LFIA: Used for rapid screening but has lower sensitivity than PCR; a negative result may require PCR confirmation, especially in high-risk patients.

#Culture for bacteria: Remains critical for obtaining viable isolates for antimicrobial susceptibility testing and serotyping, but has significantly lower sensitivity than nucleic acid amplification tests (NAATs) for many pathogens.

### 2.2 Characteristics of common respiratory viral infections

#### 2.2.1 Influenza virus

Influenza Virus can be classified into four genera: IFV A, IFV B, IFV C, and IFV D. IFV A and IFV B can spread extensively in the population, leading to seasonal epidemics. The vulnerable groups for IFV primarily include children, the elderly, pregnant women, and other individuals with low immunity. The predominant symptoms are pronounced systemic manifestations, such as fever, fatigue, cough, and aches in the head and body. Winter and spring are the peak seasons for IFV outbreaks (Yildiz et al., 2023).

Based on the variations in the surface glycoproteins hemagglutinin (HA, H1 through H18) and neuraminidase (NA, N1 through N11) of IFV A virions, it can be further categorized into different subtypes. Among these subtypes, H1N1 and H3N2 primarily infect humans, with the former two still being significant components of seasonal influenza (Zeng et al., 2024). In contrast, transmitting H5N1 and H7N9 viruses is restricted to the poultry-human interface. In most instances, avian influenza viruses infect humans with limited capacity for person-to-person transmission (Sutton, 2018). However, reports indicate that mutant strains of H5N1 and H7N9 may acquire the capacity for sustained person-to-person transmission (Plaza et al., 2024). IFV A is highly mutable and capable of generating new subtypes that may trigger global epidemics. IFV B is classified into the Victoria and Yamagata lineages. It infects fewer animal species and usually causes regional epidemics, rather than global outbreaks (Paget et al., 2022). IFV C is a relatively obscure type of IFV. Infection with it most commonly presents as URTI, which is associated with mild respiratory diseases. The clinical features and epidemiology of this subtype remain incompletely understood (Sederdahl and Williams, 2020). A study reveals that the serum positive rate of IFV C reaches up to 90% among individuals aged 7–10 years old, suggesting that most individuals are infected with IFV C at least once during childhood, and co-infection with other respiratory viruses is pretty common (Sederdahl et al., 2024). IFV D, first isolated from pigs with flu-like symptoms in 2011, is a novel virus. Currently, no direct evidence indicates that this virus causes clinical diseases in humans or spreads from person to person (Skelton and Huber, 2022).

#### 2.2.2 Respiratory syncytial virus

Respiratory Syncytial Virus has a single serotype but is divided into two subtypes, A and B, which are primarily determined by antigenic drift and duplication within the RSV-G sequence (Bergeron and Tripp, 2022). It is a prevalent cause of LRTI across all age groups globally, and is the leading cause of hospitalization for viral RTI in infants and young children.

Respiratory Syncytial Virus can cause more severe harm in premature infants, as well as those with congenital heart disease or primary immune deficiency (Fretzayas and Moustaki, 2017). Its clinical manifestations are classified into mild and severe forms: mild cases typically present as acute URTI, while severe cases manifest as LRTI, such as bronchitis and pneumonia (Tian et al., 2023). Epidemics mainly occur in winter and early spring (Szymański et al., 2025). RSV commonly causes severe respiratory disease and hospitalizations among the elderly (World Health Organization [WHO], 2023a). A multi-regional study covering 14 countries and regions revealed RSV as the primary respiratory pathogen for influenza-like illness in people over 65, and as the second most common virus leading to hospitalizations after IFV (Martinón-Torres et al., 2024). In the United States, a study of 406 respiratory tract samples found that RSV-B GB5.0.5a was the predominant genotype, while RSV-A GA2.3.5 was detected less frequently. However, patients infected with RSV-A were hospitalized more often, despite its lower detection rate (Yunker et al., 2024).

#### 2.2.3 Human rhinovirus

Human Rhinovirus belongs to the small RNA Viridae family, has one of the smallest genomes and simplest structures among RNA viruses. It can be classified into three major genotypes, A, B, and C, which accounted for 59%, 2% and 39% of the total, respectively (Zhao et al., 2016). Notably, HRV-A encompasses over 100 serotypes (Numata et al., 2023). It is the primary pathogen responsible for the common cold, accounting for approximately 50%–80% of URTI cases (Shimada et al., 2023). Since its discovery in 2006, HRV-C has been recognized as a significant pathogen contributing to severe respiratory diseases in children (Li W. et al., 2021). Studies have indicated that HRV-C infection accounts for 26.3% of pediatric intensive care unit (ICU) admissions (Galindo-Fraga et al., 2022). Another study of children with asthma using nasopharyngeal swabs reported an HRV-C detection rate as high as 68.4% (Su et al., 2020), whereas another study investigating LRTI in children with sputum found HRV-C in only 33% of cases (Ahn et al., 2018). The detection rates vary significantly across different studies, indicating that the association between HRV-C and severe LRTI in children requires careful interpretation of sampling sites. Although nasopharyngeal swabs are commonly used for HRV detection due to their non-invasive nature, positive results from the upper respiratory tract may not effectively distinguish asymptomatic colonization from LRTI (Baillie et al., 2019). Therefore, when supported by evidence from lower respiratory tract samples, the reported high incidence of severe disease caused by HRV-C more accurately reflects its pathogenic potential.

Epidemiological data suggest that infants, young children, the elderly, and immunocompromised individuals are the primary populations susceptible to HRV (Ortega et al., 2021). It circulates year-round, with infection peaks in autumn and spring (Morelli et al., 2025). HRV mainly causes URTI, while LRTI is relatively uncommon (Giardina et al., 2022). HRV can trigger cluster outbreaks in enclosed environments, such as schools and hospitals. During the 2022 COVID-19 pandemic, Japan reported an in-hospital outbreak of bronchitis caused by HRV (Shimada et al., 2023). HRV is characterized by high mutation rates and genomic diversity, resulting in a broad range of serological profiles. To date, a total of 174 serotypes have been identified (Zhao et al., 2024), presenting a substantial challenge to vaccine development. Currently, there are no approved preventive vaccines or specific antiviral medications for HRV (van den Braak et al., 2022). HRV-A and HRV-C are common HRV species; in contrast, RTI caused by HRV-B is the rarest and has milder symptoms. Related issues such as its clinical characteristics, epidemiology, and pathogenic mechanisms remain to be further studied (Georgieva et al., 2023).

#### 2.2.4 Human parainfluenza virus

Human Parainfluenza Virus is classified into four subtypes: HPIV-1 through HPIV-4. HPIV-1 and HPIV-3, belonging to the respirovirus genus, are more commonly associated with infections, and HPIV-3 infection typically presents more severely (Kurebayashi et al., 2021), particularly in high-risk populations. HPIV-2 and HPIV-4 belong to the genus Mumps virus (Sugimoto et al., 2024). HPIV predominantly causes respiratory diseases and is a significant pathogen for LRTI in children. The diseases associated with HPIV infection encompass URTI, laryngotracheobronchitis, bronchitis, bronchiolitis, and pneumonia (Wang et al., 2023b). HPIV is also a common pathogen of community-acquired RTI, mostly in children under 5 years old (Park et al., 2018). The epidemiological traits of HPIV are closely associated with its subtypes. Each subtype has distinct clinical epidemiology and clinical features, leading to different manifestations of the diseases they cause (Li Z. J. et al., 2021). HPIV-1/2 primarily infects the upper respiratory system, inducing local inflammation and edema. HPIV-3 has a greater propensity to invade the bronchi and alveoli, resulting in bronchiolitis and viral pneumonia. Owing to the rapid decline of antibodies, HPIV-3 has the highest reinfection rate. In contrast, HPIV-4 causes sporadic infections with mild symptoms and can affect individuals of all ages (Han et al., 2022; Parsons et al., 2023). Most studies indicate that HPIV-1 and HPIV-3 typically peak in summer and fall, exhibiting a distinct seasonal epidemic cycle. However, some tropical-region studies have shown an unclear epidemic season (Li J. et al., 2023), potentially due to the year-round warm local climate, which facilitates the virus’s spread. This disparity suggests that when investigating pathogen epidemic patterns, factors such as regional climate should be fully taken into account.

#### 2.2.5 Human coronavirus

Coronaviruses are the primary cause of the common cold in adults and can lead to upper respiratory infections in children. Currently, seven human coronaviruses are known to infect humans (Barthorpe and Rogers, 2022). Four (229E, HKU1, NL63, and OC43) cause mild symptoms, typically resulting in mild URTI and accounting for 15%–30% of adult common cold cases (Liu et al., 2021:428–440). The other three: Severe Acute Respiratory Syndrome Coronavirus (SARS-CoV), Severe Acute Respiratory Syndrome Coronavirus 2 (SARS-CoV-2), and Middle East Respiratory Syndrome Coronavirus (MERS-CoV), can cause severe respiratory diseases, leading to severe acute respiratory syndrome (SARS), Middle East respiratory syndrome, and COVID-19 in human history, respectively. The pathogen of SARS, a novel coronavirus variant genetically related to influenza viruses, is highly contagious. It mainly manifests as pneumonia, and some patients quickly develop symptoms like breathing difficulty, respiratory distress, and respiratory failure (Zhu et al., 2020). A survey from the United States showed that HCoV can infect all age groups, with a 29.2% incidence in those aged 0–4, 22% in those over 40, and the highest incidence rate in the 15–19 age group (Monto et al., 2020). Coronavirus infections are widespread globally and temperature-sensitive, mainly occurring in winter and early spring; however, some coronavirus outbreaks have no obvious seasonal characteristics (Rayan, 2021).

#### 2.2.6 Human metapneumovirus

Human Metapneumovirus was first discovered in 2001 and can cause URTI and LRTI across all age groups. It has been recognized as one of the top six pathogens responsible for community-acquired pneumonia (CAP) (Miyakawa et al., 2025). HMPV infection-induced symptoms are typically mild, while the elderly, children, individuals with low immune function, or those suffering from chronic respiratory diseases are susceptible to developing severe conditions after infection (Martinez-Rodriguez and Banos-Lara, 2020). In 2018, an estimated 11.1 million acute LRTI cases, notably, 502,000 hospitalizations, and 11,300 deaths worldwide were causally attributable to HMPV (Wang et al., 2021). Around 58% of hospitalized cases were infants under 12 months old, and 64% of in-hospital deaths occurred in infants under 6 months old. Of these, 79% took place in low-income and lower-middle-income countries. In China, the proportion of HMPV in the pathogen spectrum of acute respiratory infections is relatively low (Feng et al., 2024). HMPV comprises two genotypes, A and B, which can be further classified into four subtypes: A1, A2, B1, and B2 (Ye et al., 2023). These subtypes are usually prevalent simultaneously, and there are no notable differences in transmissibility or pathogenicity among them, which can cause both acute URTI (Acharya and Byrareddy, 2025) and LRTI (Miyakawa et al., 2025). HMPV is globally widespread and can cause infections throughout the year. It predominantly emerges from late winter to early spring (Lim et al., 2020) and can be transmitted simultaneously with other respiratory viruses during the high-incidence season of RTI.

### 2.3 Characteristics of common respiratory bacterial infections

#### 2.3.1 Streptococcus pneumoniae

*S. pneumoniae* is a capsulated bacterium. Its capsular polysaccharides act as a crucial virulence factor. Based on antigenic variations in these polysaccharides, *S. pneumoniae* can be categorized into 46 groups and over 100 serotypes (Kwun et al., 2022). Only a few serotypes are responsible for most infant-related diseases. These serotypes are also the most common cause of bacterial pneumonia in children. Despite declining pediatric mortality and fewer pneumonia-related deaths, *S. pneumoniae* infections remain the leading cause of death among children (Narciso et al., 2025).

Children under 5, the elderly over 65, and individuals with weakened immune systems are most susceptible to *S. pneumoniae*. Infection can cause non-invasive diseases, such as otitis media and sinusitis. It may also result in invasive pneumococcal diseases (IPD), such as pneumonia and meningitis (Narciso et al., 2025). *S. pneumoniae* is the most common cause of severe pneumonia (Monto et al., 2020), especially in people with chronic diseases. The rank of *S. pneumoniae* serotypes responsible for severe diseases varies by country. The 10 most common serotypes causing IPD are 8, 3, 22F, 12F, 19A, 9N, 7F, 15A, 33F, and 10A. Together, they account for 62% of serotype isolates (Oligbu et al., 2019). In China, common IPD serotypes are 19A, 19F, 23F, 3, and 14. For adults, the main types are 3, 19F, 19A, 23F, and 14. For children, the main types are 19A, 23F, 19F, 14, and 6B (Li M. C. et al., 2021). *S. pneumoniae* now accounts for 15% of pneumonia cases in the US and 27% of cases worldwide. In developing countries, young children and the elderly are the most vulnerable. The 7–13 serotypes included in the PCV can prevent 50%–80% of pediatric pneumococcal diseases globally (Miller et al., 2011). Currently, 139 countries have incorporated PCV into their infant immunization programs. The widespread use of PCV has reduced the burden of IPD among children under five (Reyburn et al., 2023).

With the application of *S. pneumoniae* vaccines, countries that have incorporated the *S. pneumoniae* vaccine into their national immunization programs show a reduction in the overall disease burden associated with *S. pneumoniae*, while experiencing an increase in the number of cases caused by certain non-vaccine serotypes (NVT). This increase is attributed to serotype and capsule replacement mechanisms (Micoli et al., 2023), thereby reconstituting a significant disease burden. While elevating the vaccination rate, we must remain vigilant about the rise in NVT-related diseases.

#### 2.3.2 Mycoplasma pneumoniae

*M. pneumoniae* is recognized as one of the primary pathogens responsible for CAP. It is predominantly transmitted via respiratory droplets following close contact with infected individuals. Especially among children under the age of 14, approximately 20%–40% of CAP cases can be attributed to Mp infections (Zhang et al., 2020). Estimates suggest that the global prevalence of Mp-related infections stands at 8.61% (Meyer Sauteur and Beeton, 2024), with China and Japan being notably affected regions. In the Asia-Pacific region, the infection rate of Mp varies significantly, with the highest infection rate in China (Ai et al., 2024; Chih-Cheng et al., 2024). However, during the initial phase of the COVID-19 pandemic, the adoption of non-pharmaceutical interventions led to a substantial decrease in the global detection rates of Mp and other respiratory pathogens (Huang et al., 2020).

The outbreak of Mp has seasonal and regional characteristics. It primarily occurs in autumn and winter, exhibiting a global epidemic trend. The outbreak of Mp infection in 2023 aligns with the 4-year epidemic cycle. The post-pandemic epidemic season began in August, consistent with the pre-pandemic situation (Li Q. et al., 2024). *Mycoplasma pneumoniae* pneumonia (MPP) is self-limiting. Most patients have a favorable prognosis, but a small proportion may progress to severe pneumonia. Thus, early detection and appropriate treatment are crucial to prevent deterioration. For treatment, β-lactam antibiotics are ineffective, as Mp lacks a cell wall. Effective drugs mainly include macrolides, tetracyclines, or quinolones, which inhibit protein or DNA synthesis (Wang et al., 2024). With the increasing use of macrolides, macrolide-resistant *Mycoplasma pneumoniae* (MRMP) has become a widespread global issue since 2000. Globally, infection rates associated with MRMP are rising and vary regionally. The Western Pacific region has the highest infection rate, at 53.4%, followed by Southeast Asia at 9.8%, the Americas at 8.4%, and Europe at 5.1% (Kim et al., 2022). Research has shown a correlation between MRMP and both the symptoms and the duration of fever in patients (Pereyre et al., 2016). For MPP patients who are treated with macrolides but do not improve, switching to second-line medications such as tetracyclines or fluoroquinolones may be prudent (Cai et al., 2024).

#### 2.3.3 Haemophilus influenzae

*H. influenzae* is a pleomorphic, Gram-negative coccobacillus that can cause various airway mucosal infections and invasive diseases, including bacterial meningitis. The capsule is a crucial virulence factor. Based on the presence of the capsule, *H. influenzae* can be categorized into encapsulated and non-encapsulated forms. The encapsulated type can be further classified into six serotypes, A through F. In contrast, the non-encapsulated type is collectively termed Non-typeable *Haemophilus influenzae* (NTHI) and does not agglutinate with any of the classified serotypes (Oliver et al., 2023). *Haemophilus influenzae* type B (Hib) ranks as the second most common causative agent of bacterial pneumonia. However, since the introduction of the Hib vaccine in the 1990s, the number of Hib cases and their incidence rate have declined. As a result, the disease burden associated with *H. influenzae* has increasingly been dominated by NTHI (Takla et al., 2020). Particularly among children with compromised immune systems, NTHI-related diseases tend to be more severe (Gozum et al., 2020). Specifically, NTHI is a mucosal pathogen specific to humans. When colonizing the nasopharynx, extensive DNA fragment exchanges readily occur among NTHI strains, allowing them to acquire novel virulence factors and thereby enhance their invasiveness. Furthermore, NTHI is the primary causative agent of acute otitis media in children, as well as acute sinusitis, pneumonia, and conjunctivitis. Moreover, it is also a significant pathogen in adult patients with chronic obstructive pulmonary disease (COPD) (Landwehr et al., 2024). Chronic NTHI infection can exacerbate airway inflammation, and colonization by NTHI may lead to the deterioration of COPD (Short et al., 2021). It is important to note that, in contrast to Hib, NTHI strains seldom invade the bloodstream and cause systemic infections. Notably, during the COVID-19 pandemic, the incidence of invasive diseases caused by *H. influenzae* decreased significantly in various countries and regions; this may be associated with the implementation of COVID-19 control measures (Brueggemann et al., 2021). Tsang et al. (2017) conducted an investigation into the antimicrobial resistance of different *H. influenzae* types. Their findings revealed that the positive rate of β-lactamase in NTHI strains was more than twice that in encapsulated strains. This observation implies a correlation between the substantial rise in ampicillin resistance among *H. influenzae* and the prevalence of NTHI.

#### 2.3.4 Klebsiella pneumoniae

*K. pneumoniae* is a facultative anaerobic, Gram-negative enterobacterium that commonly colonizes the upper respiratory and digestive tracts. When the host’s immune system fails to regulate the growth of this pathogen, the colonization state can progress to an infectious state. Among *Klebsiella* species, the diseases caused by *K. pneumoniae* are the most prevalent, resulting in various infections, including CAP, sepsis, and meningitis. *K. pneumoniae* is also one of the primary pathogens responsible for clinical infections. It can trigger outbreaks of nosocomial infections, particularly Ventilator-associated Pneumonia (VAP) in patients undergoing mechanical ventilation. Based on the long-term trends in the isolation rates of major Gram-negative bacilli, it was discovered that in recent years, the overall isolation rate of *K. pneumoniae* specimens has consistently ranked second only to that of *Escherichia coli*. Moreover, *K. pneumoniae* has the highest isolation rate among all pathogens in RTI (Hu et al., 2022). Based on its virulence characteristics, *K. pneumoniae* can be classified into classic *Klebsiella pneumoniae* (cKP) and hypervirulent *Klebsiella pneumoniae* (hvKP), with hvKP being the highly virulent variant of *K. pneumoniae* (Choby et al., 2020). HvKP not only causes infections in patients with compromised immune function but also leads to community-acquired infections in young individuals with normal immune status. Moreover, it is more susceptible to triggering severe invasive and disseminated infections. *K. pneumoniae* is primarily serotyped according to its capsular polysaccharide (K antigen) and lipopolysaccharide (LPS) O antigen. The K antigen is a crucial factor contributing to the enhanced virulence of *K. pneumoniae* (Zhang et al., 2020). K1 and K2 are the predominant serotypes of hvKP (Huang et al., 2015). The mucinous phenotype of *K. pneumoniae* is associated with its capsule. The highly mucinous characteristic of hvKP renders it more susceptible to causing invasive infections. Based on the differences in O antigens, *K. pneumoniae* can be classified into nine serotypes, with O1 and O2a being the two most prevalent types (Lipworth et al., 2021). Carbapenem-Resistant *Klebsiella Pneumoniae* (CRKP) and hvKP strains exhibit distinct serotype distributions. Among CRKP strains, O2a: K14K64 (41.0%) was the most frequently observed serotype, whereas among hvKP strains, O1: K1 (26.4%) and O1: K2 (17.3%) were the predominant serotypes (Zhang et al., 2020).

Historically, it has been commonly assumed that hvKP has a low drug-resistance rate. However, recent research indicates that hvKP infections demonstrate extensive drug resistance (Pu et al., 2023). The virulence factors of hvKP, including capsules and virulence plasmids, may contribute to its drug-resistance mechanism. Specific serotypes (e.g., K1 and K2) not only possess high virulence but also exhibit resistance to certain antibiotics (Li L. et al., 2023). Notably, the resistance rates of hvKP to carbapenem antibiotics have increased substantially, with the prevalence of CRKP exceeding 20%. CRKP is typically associated with hospital-acquired urinary tract infections, pneumonia, sepsis, and soft-tissue infections. Due to the lack of effective antibacterial therapies, the outbreaks and rapid dissemination of CRKP in hospitals have become a significant threat to public health. In the early stage of *K. pneumoniae* development, HvKP and CRKP emerged as distinct entities. However, currently, the demarcation between these two types of infections is blurring. Carbapenem-resistant and hypervirulent *Klebsiella pneumoniae* (CR-hvKP), a strain that combines high virulence with multidrug resistance, was first reported in 2018 (Gu et al., 2018). The emergence of CR-hvKP is particularly alarming, as it simultaneously exhibits multidrug resistance, high virulence, and high infectivity (Dai and Hu, 2022; García-Cobos et al., 2025), it has evolved into a global public health concern.

#### 2.3.5 Pseudomonas aeruginosa

*P. aeruginosa* is a non-fermentative Gram-negative bacillus. It is widely distributed in nature and hospital environments. *P. aeruginosa* is known for its easy colonization, genetic plasticity, multidrug resistance, and biofilm formation (Jurado-Martín et al., 2021). As an opportunistic pathogen, it is a leading cause of hospital-acquired infections. It mainly results in acute and chronic LRTI, including pneumonia and bronchiectasis. *P. aeruginosa* is especially common in hospital-acquired pneumonia (HAP) and VAP (Zaragoza et al., 2020). This bacterium secretes numerous virulence factors, including LPS, the type VI secretion system, pyocyanin, elastase, alkaline protease, and biofilms. These factors aid infection and worsen disease conditions (Vidaillac and Chotirmall, 2021). Although rare in CAP (Chen Y. et al., 2025), *P. aeruginosa*-induced CAP occurs in 67.0% of patients with a history of *P. aeruginosa* infection, bronchiectasis, or very severe COPD (Restrepo et al., 2018). *P. aeruginosa* accounts for 16.9%–22.0% of HAP cases in China, second only to Acinetobacter baumannii (Chen et al., 2023). The in-hospital mortality rate for *P. aeruginosa*-caused HAP can reach 40.1% (Micek et al., 2015). *P. aeruginosa* in hospital infections shows high drug resistance. It has natural resistance to common antibiotics, complicating treatment. Extensive carbapenem use has increased *P. aeruginosa* drug resistance, leading to carbapenem-resistant *Pseudomonas aeruginosa* (CRPA). In 2017, the WHO identified CRPA as a critical pathogen (Tacconelli et al., 2018). Recently, multidrug-resistant *Pseudomonas aeruginosa* (MDR-PA) has increased globally, posing a public health threat. *P. aeruginosa* shows both intrinsic and acquired multidrug resistance. In HAP, MDR-PA rates are high, and CRPA proportions range from 36.6% to 44.8%. Among VAP patients, MDR-PA rates are even higher (Dong et al., 2025).

### 2.4 Characteristics of common respiratory fungal infections

#### 2.4.1 Aspergillus

*Aspergillus* is a globally widespread filamentous fungus commonly found in natural environments such as air, water, and soil (Dladla et al., 2024). Among its subspecies, *Aspergillus fumigatus* is the most prevalent and pathogenic (Janssens et al., 2024). In healthy individuals, inhalation of spores rarely causes pulmonary disease, but it can lead to severe infections in immunocompromised patients (Moldoveanu et al., 2021). Aspergillosis exhibits global distribution with rising incidence in recent years (Denning, 2024). In Indonesian ICU patients with invasive pulmonary aspergillosis, *Aspergillus flavus, Aspergillus fumigatus*, and *Aspergillus niger* constitute the three predominant causative species (Rozaliyani et al., 2021). In a cross-sectional study from Iran, *Aspergillus flavus* was also the predominant species within the *Aspergillus genus* (Badiee et al., 2022). Conversely, a Chinese research identified *Aspergillus fumigatus* as the primary pathogen, followed by *Aspergillus niger* and *Aspergillus flavus* (Bilal et al., 2023). *Aspergillus* is the most common fungal pathogen in VAP, accounting for 8% of all VAP cases (Fortún, 2022). In immunocompromised patients, invasive aspergillosis is a significant cause of morbidity and mortality (Cadena et al., 2021).

Aspergillus infections are opportunistic and species-specific, with the vast majority of severe infections occurring in individuals with compromised immune systems or underlying lung disease. Immunodeficiency, allergy, and prior pulmonary disease are the most significant risk factors, with clinical manifestations determined by microbial and host-specific factors (Janssens et al., 2024). Given Aspergillus’s ubiquitous environmental presence, its isolation from respiratory specimens may indicate colonization, allergy, or infection, necessitating comprehensive evaluation incorporating clinical presentation, imaging, and laboratory findings. Antifungal therapy is the primary treatment for aspergillosis (Salzer, 2023). Azole antifungals are the first-line choice, widely used for their efficacy and tolerability. However, some patients respond poorly to treatment, and antifungal therapy may further impair lung function, particularly when fungal nodules form (Chirumbolo et al., 2024). Chronic pulmonary aspergillosis may require long-term antifungal treatment (Armstrong-James et al., 2023).

#### 2.4.2 Candida

*Candida* species commonly colonize the respiratory tract but may become opportunistic pathogens under specific conditions. *Candida*, a globally distributed organism, causes many hospital-acquired infections. Hospitals most frequently isolate *Candida albicans*, though detection rates of non-albicans *Candida* have increased recently (Gülmez et al., 2021). ICU patients show much higher detection rates of *Candida* in respiratory specimens than those in general wards (Glück et al., 2025), possibly because broad-spectrum antibiotics are often used in ICU. Critically ill patients on mechanical ventilation frequently experience lower respiratory tract colonization by *Candida* (Azoulay et al., 2006).

*Candida* pneumonia is rare and usually affects patients with severe underlying conditions. The primary disease or other infections often mask its clinical signs. Confirming primary candidal pneumonia almost always means extreme immunosuppression and high mortality. Pneumonia from disseminated candidemia, where *Candida* spreads from the blood to the lungs, has a mortality rate over 40%, rising in those with disseminated infection, ICU admission, or immunosuppression (Kazancioglu et al., 2024; Imoto et al., 2025). Diagnosis needs rigorous evaluation of risk factors, microbiology, and pathology. *Candida* in respiratory samples usually indicates colonization: a positive result alone does not confirm infection but helps guide identification and susceptibility testing. *Candida* blood cultures with pulmonary infiltrates strongly indicate hematogenous disseminated pneumonia (Chen et al., 2018). Do not treat respiratory colonization. Start antifungal therapy immediately if infection is diagnosed or strongly suspected (Ahmed et al., 2025). Manage respiratory *Candida* infection with a comprehensive assessment and integrate histopathology and microbiology for diagnosis. Tailor antifungal therapy to the case and monitor for resistance (Cornely et al., 2025).

#### 2.4.3 Mucorales

Mucorales, including *Rhizopus, Mucor, and Lichtheimi*, are widespread in the environment and harmless to healthy individuals (Skiada et al., 2025). In immunocompromised patients, however, they can cause severe, invasive diseases, most often affecting the lungs. Respiratory mucormycosis is rare but usually fatal (Panda et al., 2024). *Mucor* species exhibit rapid, aggressive progression and high mortality (Banimostafavi et al., 2022). Globally, mucormycosis incidence is <0.06 cases per million in developed countries, increasing with more immunosuppressed patients (Skiada et al., 2024). Mortality ranges from 40% to 80% (Motamedi et al., 2022; Skiada et al., 2024). In China, pulmonary mucormycosis is most common, often linked to diabetes, and 52.5% have a poor prognosis (Qu et al., 2021). Among lung transplant recipients, incidence is <1%, but mortality nears 100% (Fernández González et al., 2025). Diagnosis and treatment remain difficult, as imaging is non-specific and mimics *Aspergillus*. Histopathology is the diagnostic standard; biomarkers are insensitive (Czech and Cuellar-Rodriguez, 2025). Plasma cfDNA PCR is highly sensitive and specific for immunosuppressed patients (Mah et al., 2025). Early intervention improves outcomes; amphotericin B is the first-line treatment, as Mucorales generally resist most azoles (Ganesan et al., 2022). Though rare, these infections are a serious threat due to aggressiveness, diagnostic challenges, and limited therapies. Early confirmation depends on integrating microbiology, molecular tests, pathology, managing underlying conditions, and multidisciplinary care (Ganesan et al., 2022).

#### 2.4.4 Pneumocystis

*Pneumocystis jirovecii* is an obligate extracellular fungus with unique biological characteristics. As an atypical fungus, it spreads through airborne droplets and parasitizes the pulmonary parenchyma of various mammals (Hauser, 2021). In immunocompetent humans, it usually causes asymptomatic colonization or mild symptoms. In immunocompromised individuals, it can progress to severe, life-threatening *Pneumocystis* pneumonia (PCP) (Ames et al., 2023). PCP occurs worldwide. It mainly results from activation of latent infections but can also spread through person-to-person transmission, especially in hospitals where immunocompromised patients gather (Charpentier et al., 2017).

Classification and phylogenetic studies reveal that *Pneumocystis* exhibits high host specificity across mammalian hosts (Cisse et al., 2021). In humans, *Pneumocystis jirovecii* is a common pathogen causing severe pneumonia in HIV/AIDS patients (Kolbrink et al., 2022). In Africa, *Pneumocystis jirovecii* exhibits higher colonization and infection rates among HIV-positive adults, though epidemiological data remain insufficient (Wills et al., 2021). However, its incidence is relatively elevated in non-HIV immunocompromised patients, such as those with organ transplants, autoimmune diseases, or hematologic malignancies (Barreto et al., 2016). Mortality rates remain high even with treatment. The mortality rate for HIV-associated PCP is approximately 10%–20%, while non-HIV immunosuppression-related PCP carries a higher mortality rate of 30%–50% (Lemiale et al., 2025). This disparity may stem from HIV patients having greater access to early diagnosis and treatment, whereas non-HIV patients often experience a more abrupt onset and faster disease progression. In 2022, *Pneumocystis jirovecii* was designated by the WHO as a priority fungal pathogen, underscoring its public health significance (Ma et al., 2025). Research into its epidemiology, host specificity, and genetic diversity provides crucial foundations for prevention and control. Concurrently, early diagnosis and targeted treatment are paramount for improving patient outcomes.

## 3 Clinical predicaments in the treatment of RTI

### 3.1 Overlapping symptoms of RTI pose significant diagnostic challenges

The diagnosis and treatment of RTI face numerous challenges. The pathogens are highly diverse, and infections caused by different pathogens often present a high degree of similarity in symptomatic presentation. Additionally, the clinical manifestations of an infection caused by the same pathogen can vary significantly among different individuals or at different stages of the disease. For instance, in cases of IFV infection, some patients may only experience mild upper respiratory tract symptoms. In contrast, others may rapidly progress to severe pneumonia and develop serious complications such as respiratory failure. Additionally, a single clinical symptom, such as a cough, can stem from multiple causes, including viral infections, bacterial infections, or allergies. Although imaging features may offer some clues, their specificity is inadequate. It is challenging to accurately differentiate infections caused by different pathogens based solely on clinical manifestations and epidemiological characteristics.

### 3.2 Treatment challenge - the abuse of antibiotics

In recent years, with the increasing use of broad-spectrum antibiotics in clinical practice, notable changes have occurred in the drug resistance profiles and the composition of pathogenic bacteria. The continuous emergence of multidrug-resistant bacteria has presented substantial challenges to clinical treatment. Relevant data indicate that between 1990 and 2021, over one million people died annually from antibiotic-resistant bacterial infections worldwide (GBD 2021 Upper Respiratory Infections Otitis Media Collaborators, 2024). It is estimated that by 2050, 82.2 million people globally will succumb to such infections (GBD 2021 Antimicrobial Resistance Collaborators, 2024). Due to the challenges in accurately identifying specific pathogens, empirical treatment is commonly employed in the clinical management of RTIs. This approach results in issues such as poor treatment specificity and a high likelihood of antibiotic abuse. Currently, the unreasonable use of empirical antibiotics for RTI is widespread. Between October 2014 and April 2018, among 173 million outpatients and emergency patients in 139 hospitals in China, antibiotic prescriptions accounted for 10.9% (approximately 18.85 million prescriptions). Over 60% of pneumonia patients in outpatient and emergency departments were prescribed antibiotics. Among these prescriptions, the proportion of irrational ones was as high as 51.4% (Zhao et al., 2021).

### 3.3 Co-infection of pathogens leads to synergistic morbidity

Respiratory pathogen co-infection refers to the situation in which a single host is infected with two or more respiratory pathogens either simultaneously or sequentially. The synergistic effect of co-infection poses greater challenges to clinical diagnosis and treatment. Studies using animal models of respiratory tract co-infection have demonstrated that pathogen-pathogen interactions can exacerbate the disease (Rüger et al., 2021). In human cases, co-infection with COVID-19 and *S. pneumoniae* can lead to synergistic pulmonary inflammation (Barman et al., 2022). When COVID-19 co-occurs with tuberculosis, the presence of similar symptoms such as fever and cough can easily result in misdiagnosis (Daneshvar et al., 2023). Co-infection with IFV and Aspergillus significantly elevates mortality rates (Roe, 2023). In children with Mp co-infected with respiratory viruses, the duration of fever is longer, and such co-infection is strongly associated with refractory pneumonia in children (Choo et al., 2022). Mechanistic studies have revealed potential immunomodulatory effects among pathogens. Co-infection with mycoplasma and viruses may exacerbate immune dysregulation by inhibiting the interferon pathway (Mochan and Sego, 2023). In summary, respiratory pathogen co-infection is a complex, multi-factorial phenomenon. Clinical management of such cases necessitates a comprehensive assessment that integrates epidemiology, etiological detection, and the host’s immune status.

### 3.4 Prevention and vaccine bottlenecks

The respiratory tract’s distinctive physiological structures, including the mucus layer and ciliary movement, present natural barriers to vaccine delivery. While mucosal immunity can offer local protection, it is challenging to elicit a long-lasting immune response. Although vaccines such as those for influenza, pneumococcus, and Hib are widely used and have effectively curbed the spread of respiratory diseases, a lack of effective vaccines remains for pathogens such as HRV, HPIV, and metapneumovirus. Although advancements have been made in RSV vaccines, they still encounter bottlenecks in terms of both the duration of protection and coverage (Li Q. et al., 2024). Existing vaccines primarily target single pathogens, offering limited broad-spectrum protection. Certain respiratory viruses, such as the IFV and the novel coronavirus, replicate at a rapid pace and can spread extensively before adaptive immunity is formed. This phenomenon also shortens the effective window for vaccine intervention (Livieratos et al., 2024). The high-frequency mutations of viruses necessitate frequent vaccine updates (Sayers, 2023), which restricts the broad-spectrum protective efficacy of current vaccines, vaccine coverage is still inadequate.

**FIGURE 1:**
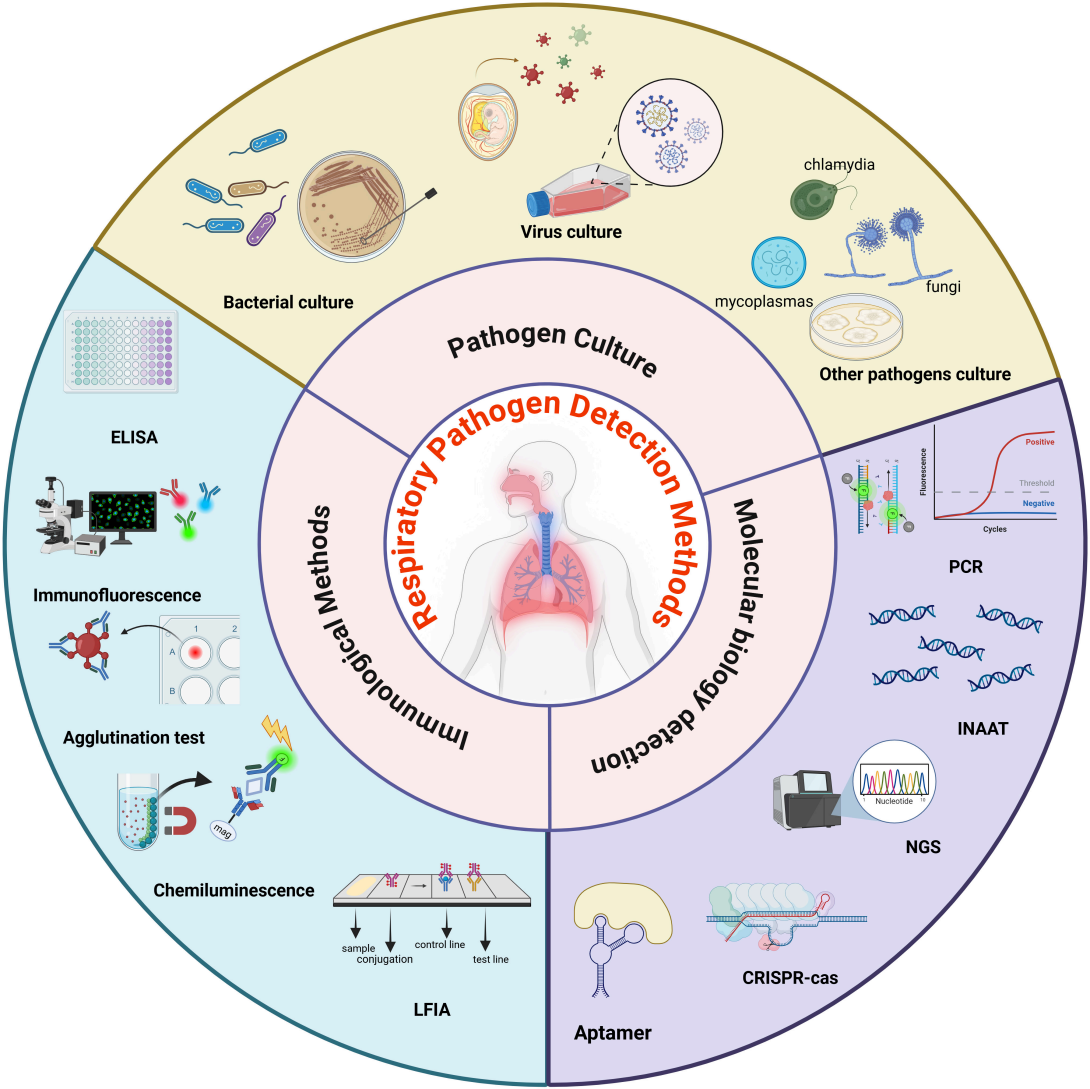
The summary of respiratory pathogen detection methods. Respiratory pathogen detection methods are primarily categorized into three main types: pathogen culture, immunological detection, and molecular biological detection. Pathogen culture primarily encompasses bacterial culture, viral culture, and other types of pathogen cultures. Immunological detection methods primarily include ELISA, immunofluorescence assays, agglutination tests, chemiluminescence assays, and LFIA. Molecular biological methods primarily include PCR and its derivatives, INAAT, next-generation sequencing, CRISPR/Cas, and aptamers. ELISA, enzyme-linked immunosorbent assay; LFIA, lateral flow immunoassay; PCR, polymerase chain reaction; INAAT, isothermal nucleic acid amplification techniques; CRISPR/cas, clustered regularly interspaced short palindromic repeats/associated systems (Image created with BioRender.com, with permission).

In conclusion, selecting an appropriate detection method for respiratory pathogens is crucial for the accurate diagnosis and effective treatment of respiratory infections. Currently, the main clinical detection methods for respiratory pathogens include pathogen culture, immunological methods, and molecular biology detection (Figure 1). However, due to the limitations of existing technologies, especially in the detection of CAP, approximately 40% of pathogens remain unidentified (van Ettekoven et al., 2024). Currently, there is no single detection technology that combines high sensitivity and specificity to enable rapid and efficient differential diagnosis of pathogens. There is still significant scope for improvement in the accuracy and effectiveness of laboratory diagnosis. Advantages, disadvantages, clinical applications and performance of common respiratory pathogen detection methods are summarized in Table 2. We will discuss these detection methods in the following.

**TABLE 2:** Advantages, disadvantages, clinical applications and performance of common respiratory pathogen detection methods.

Detection methods	Advantages	Disadvantages	Clinical applications	Performance evaluation testing time	References
Pathogen culture	Low cost, direct pathogen detection, “gold standard,” and can provide drug sensitivity test information	Long testing cycles and susceptibility to antimicrobial agents may limit its sensitivity; low positive rate, with only a small proportion of pathogens successfully cultured; high personnel requirements.	Common respiratory bacteria cultures are usually combined with other rapid detection methods such as molecular and mass spectrometry to compensate for deficiencies	Sensitivity: >40%; specificity: almost 100%; testing time: 18–24 h, and even longer	Gajic et al., 2022; Aoki et al., 2021; Lin et al., 2023; Wang et al., 2023e; Wang H. et al., 2019
Enzyme-linked immunosorbent assay	Easy to operate, highly specific, and can be used for quantitative analysis	Reagent-dependent, can be costly, has potential for cross-reactivity, and has a relatively long detection time	The detection of antibodies produced by respiratory pathogens, is applicable to retrospective diagnosis and epidemiological investigation	Sensitivity:>65%; specificity:>97%; testing time: 3–6 h	Yang et al., 2020; Hu et al., 2023; Claudet et al., 2025; Ramirez et al., 2023; Park et al., 2025; Horn et al., 2022; Jiao et al., 2023
Immunofluorescence	High specificity, high sensitivity, relatively rapid and straightforward, and can be analyzed in a targeted manner	Requires fluorescence microscopy, results can be subjective and require experienced personnel	It is often used to detect atypical respiratory pathogens, and has been applied in clinical detection of RSV, IFV, parainfluenza types 1-3, adenovirus, etc	Sensitivity: >70%; specificity: >95%; testing time: <4 h	Lam et al., 2021; Liu M. et al., 2023; Qin et al., 2020; Fourie et al., 2023 Tan et al., 2021
Agglutination tests	Rapid and low-cost	Easy to false positives, limited sensitivity	It is used for the diagnosis of *MP* infection, and two short-term serological IgM tests are commonly used to confirm *MP* infection at an early stage	Sensitivity: >82%; specificity: >95%; testing time: <4 h	Liu M. et al., 2023; Jeon et al., 2021; Fourie et al., 2023; Kruse et al., 2021
Chemiluminiscence	Easy to operate, high sensitivity, quantitative detection, and stable results at the early stage	High cost, with the potential for false positives or false negatives	Detect respiratory pathogen antigens or antibodies	Sensitivity > 90%; specificity: >99%; testing time: <1 h	Liu M. et al., 2023; Tiwari and Dhoble, 2018; Mekonnen et al., 2021; Wu et al., 2023
Lateral flow immunoassay	Easy to operate, fast, no special equipment required	Limited sensitivity, restricted detection range, and significantly influenced by sampling	Detection of influenza A, influenza B, RSV, MP antigen or antibody, etc.	Sensitivity: >66%, the improved method exhibits higher sensitivity; specificity: >95%; testing time: <30 min	Ding et al., 2017; Huang et al., 2016; Miller et al., 2020; Liu et al., 2025
Polymerase chain reaction	Fast, highly sensitive and specific, not affected by antimicrobial drugs	High cost, limited detection range; unable to detect novel or unexpected pathogens	widely applied and can be routinely used to detect respiratory pathogens	Sensitivity > 90%; specificity: >95%; testing time: 1–4 h	Gadsby et al., 2016; Kazuya et al., 2020; Osborne et al., 2025; Brown et al., 2024
Isothermal nucleic acid technology	Fast, highly sensitive and specific, not affected by antimicrobial drugs, low instrument requirements	Primer design is complex, prone to contamination leading to false positives, multiplexing can be challenging, and product analysis is limited	Rapid screening for respiratory pathogens, suitable for primary healthcare facilities or rapid on-site testing	Sensitivity: comparable to PCR; specificity: lacks the specificity to distinguish single nucleotide differences; testing time: <1 h	Hayashida et al., 2023; Tan et al., 2025; Park, 2022; Srivastava and Prasad, 2023
Metagenomic next-generation sequencing	Hypothesis-free detection of all nucleic acids in a sample; potential to detect virulence and antimicrobial resistance genes	High cost, human nucleic acid interference reduces sensitivity, and susceptibility to environmental microorganisms increases false positives, and the results are complicated to interpret	Critically ill respiratory infections, infections in immunocompromised patients, infections with novel or rare pathogens that yield negative results with conventional testing, and mixed infections	Sensitivity: >70%; specificity: >85%; testing time: <24 h	Diao et al., 2022; Oechslin et al., 2018; Ye et al., 2025; He et al., 2022; Miao et al., 2018; Tan et al., 2024
Targeted next-generation sequencing	Faster turnaround time and lower cost compared to mNGS, reduced host background interference through targeted enrichment and Enhanced sensitivity for pre-defined targets	Low rate of difficult-to-break microorganisms, complex operation procedures, and prone to contamination	Patients with non-severe respiratory infections who have negative results from conventional tests or fail to respond to empirical treatment, and are suspected of having specific pathogen infections	Sensitivity: >82%; specificity: >90%; testing time: <16 h	Huang et al., 2024; Zhang et al., 2024; Fourie et al., 2023; Yin et al., 2024; Gu et al., 2025

## 4 Detection methods for RTI

Accurate and timely detection of respiratory pathogens is critical for clinical management, infection control, and antimicrobial stewardship. No single method offers universal advantages. Each has distinct strengths and limitations in turnaround time, sensitivity, specificity, throughput, and cost. Clinical selection must consider the suspected pathogen spectrum, disease severity, required turnaround time, and available resources. The next section reviews three diagnostic technology categories: pathogen culture, immunological testing, and molecular biology methods. It assesses their clinical utility and evolving roles in modern laboratory medicine.

### 4.1 Pathogen culture

Pathogen culture covers bacterial, viral, and fungal cultures. It is the gold standard for pathogen detection and is central to diagnosing infectious diseases. The primary advantage is the ability to obtain viable isolates, which enables definitive identification and supports antimicrobial susceptibility testing (Gajic et al., 2022). Pathogen culture enables the direct observation and isolation of colonies, but it also presents common challenges. It requires days to weeks, detects only about 50% of lower respiratory pathogens (Aoki et al., 2021), and is vulnerable to interference from prior antibiotic use. Some pathogens are constrained by laboratory limitations or need strict growth conditions that prevent *in vitro* culture.

Numerous bacteria associated with respiratory infections exhibit strict growth requirements: some strains require specific media to provide essential nutrients, while others necessitate specialized culture conditions. Viruses and atypical pathogens, due to unique biological characteristics such as a lack of cell walls or obligate intracellular parasitism, demand highly specialized cultivation. Common methods include chick embryo culture, cell culture, and organ culture (Hu Z. et al., 2025). Currently, chick embryo culture is primarily used for vaccine production, with its application in laboratory diagnostics gradually declining (Peck et al., 2021). Virus isolation typically requires specific cell lines, such as MDCK cells for influenza viruses and A549 cells for RSV (Meng et al., 2020; Loginova et al., 2021). Obligate intracellular pathogens, such as *Chlamydia pneumoniae*, must be inoculated onto specific cell lines, including HEp-2 or HL cells (Lewis et al., 2021). Due to the high operational difficulty, these methods require specialized technicians and specific laboratory conditions, limiting their large-scale application. Currently, virus and atypical pathogen isolation and culture are primarily used in basic research and have not become routine clinical diagnostic methods. Fungal culture serves as the “gold standard” for diagnosing respiratory fungal infections. Traditional fungal culture relies on symptom observation, morphological identification, and biochemical analysis. While cost-effective, this method is time-consuming, has limited sensitivity, and is influenced by fungal growth conditions and operator expertise (Chen et al., 2018). These limitations are particularly pronounced in high-risk populations such as immunocompromised patients and those with chronic respiratory diseases. To overcome these limitations, clinical practice often employs detection of fungal cell wall components in blood or bodily fluids for infection diagnosis, such as β-D-glucan (G-detection), galactomannan (GM-detection), and Candida mannan detection (Lass-Florl et al., 2021; Mercier et al., 2021). In summary, while culture techniques remain indispensable for providing antibody detection, their limitations in speed and sensitivity confine them to an auxiliary role in acute diagnosis. In clinical practice, culture techniques are generally used to substantiate, verify, or provide additional context to the findings of rapid molecular tests, rather than serving as the primary diagnostic tool for acute infections.

In clinical practice, traditional Antimicrobial Susceptibility Testing (AST) methods, such as broth dilution, disk diffusion, remain essential and reliable tools, albeit with lengthy turnaround times. The integration of novel techniques is transforming the landscape. Matrix-Assisted Laser Desorption/Ionization Time-of-Flight Mass Spectrometry enables swift and direct analysis (Wang et al., 2023d), while Next-generation sequencing (NGS) enhances the detection of pathogens and their resistance genes (Lin et al., 2023). However, these technologies have not yet supplanted phenotypic methods. Thus, AST strategy should be tailored to the clinical scenario, laboratory conditions, and the need for resistance monitoring.

### 4.2 Immunological methods

Immunological methods detect pathogen-specific antigens or host antibodies, providing rapid, cost-effective tools for diagnosis and seroepidemiology. Compared to molecular assays, immunological methods offer greater speed and simplicity, though they are generally less sensitive. These characteristics make them invaluable for point-of-care testing (POCT), outbreak screening, and retrospective serological diagnosis.

#### 4.2.1 Enzyme-linked immunosorbent assay (ELISA) and chemiluminescent immunoassay (CLIA)

Enzyme-linked immunosorbent assay and its more advanced counterpart CLIA serve as high-throughput quantitative detection platforms suitable for identifying specific antibodies, such as IgM, IgG, IgA, in serum. IgM, IgG, and IgA are different types of antibody proteins, each with distinct roles in the immune response. This technology is widely used for serological diagnosis of pathogens such as Mp (Claudet et al., 2025) and for evaluating vaccine responses (Dutt et al., 2024). It offers advantages including high specificity, high throughput, and quantitative analysis capabilities, making it suitable for large-scale screening (Zhou C. et al., 2021). However, its sensitivity has limitations: false-negative results may occur when pathogen loads in respiratory samples are low (Ghoshdastidar et al., 2020). To address this issue, ongoing improvements to ELISA technology focus on enhancing sensitivity, enabling simultaneous detection of multiple pathogens, and developing automated rapid testing methods (Yang et al., 2020; Hu et al., 2023). ELISA holds both research and clinical value in the diagnosis of respiratory pathogens, and optimizing its performance remains a key research focus. In practice, CLIA overlaps significantly with ELISA in application scenarios; however, due to its broader dynamic range of detection and higher sensitivity, CLIA has become the mainstream automated technology in clinical laboratories. In the detection of *S. pneumoniae*, its specificity (92.3%) significantly outperforms agglutination tests (Liu M. et al., 2023). Furthermore, Monitoring changes in catalytic enzyme activity to detect bacterial viability significantly shortens response times following antibiotic treatment (Zhuang et al., 2025), overcoming limitations of culture-based methods. In addition, the integration of nanoparticles, tiny particles sized between 1 and 100 nm, with chemiluminescence technology, which involves the emission of light during a chemical reaction, substantially enhances signal amplification, lowering detection limits to trace levels and dramatically improving sensitivity (Liu et al., 2016). Studies demonstrate that nanoparticle-based chemiluminescent real-time imaging exhibits high sensitivity in respiratory virus infection models. Despite these major advances in nanoparticle-enhanced chemiluminescence, most research remains in the validation phase (Tiwari and Dhoble, 2018).

#### 4.2.2 Immunofluorescence assay (IFA)

Immunofluorescence assay is a highly specific method for detecting antibodies against atypical pathogens and respiratory viruses. This technique enables visualization of antigen-antibody complexes under a fluorescence microscope. In patients with CAP, IFA is used as an auxiliary diagnostic tool to detect IgM antibodies against major respiratory pathogens in serum or plasma. This technique is recognized as effective for diagnosing atypical pneumonia and has been approved by the State Food and Drug Administration (Qin et al., 2020). Currently, it is used in clinical practice to detect RSV, IFV, parainfluenza types 1–3, adenovirus, and other pathogens (Tan et al., 2021). This method has high specificity, even the specificity of the antibody against the SARS-CoV-2 N protein reaches 100% (Lam et al., 2021).

During the COVID-19 pandemic, IFA could be used to compare the changing epidemic trends of respiratory viruses (Wan et al., 2023). However, its need for experienced personnel and specialized equipment, along with the subjectivity of interpreting results, limits its routine use. Consequently, in situations requiring high sensitivity, molecular detection methods are gradually replacing IFA (Liu M. et al., 2023).

#### 4.2.3 Agglutination tests

Agglutination tests include cold agglutination tests and passive particle agglutination (PPA) tests. The former, a traditional method with lower specificity, has been gradually phased out (Sun et al., 2025). PPA is a simple, low-cost, rapid diagnostic method. Its principle involves the formation of visible agglutination when target antigens are present, causing coated antibody particles to aggregate. PPA is commonly used for the rapid screening of respiratory pathogens, making it particularly suitable for primary healthcare settings (Wu et al., 2018). Lv et al. (2022) used PPA for large-scale Mycoplasma screening in 81,131 patients with respiratory infections. Compared to culture and ELISA methods, its operational simplicity offers marked advantages (Lv et al., 2022). While offering greater ease of use than culture and ELISA, PPA exhibits lower sensitivity (Sun et al., 2025). An improved version utilizes 3D (Three Dimensional) printing technology to optimize capture efficiency, enabling the effective extraction of antigens from large-volume samples and enhancing detection sensitivity (Wang et al., 2023a). New technologies are driving the evolution of agglutination tests from qualitative to quantitative analysis and from single-marker to multiplex detection. However, microscopic observation remains indispensable as a foundational validation method, particularly when evaluating samples that are weakly agglutinating.

#### 4.2.4 Lateral flow immunoassay (LFIA)

Lateral flow immunoassay serves as the foundational technology for rapid POCT, delivering results within minutes with minimal training and no equipment required. This technique is widely used for the detection of antigens for influenza, RSV, and SARS-CoV-2 (Huang et al., 2016; Miller et al., 2020). Traditional LFIA employs gold nanoparticles as chromogenic markers, but suffers from limitations such as low sensitivity and high false-negative rates (Tong et al., 2023). Some LFIA tests may cross-react with other respiratory pathogens, significantly limiting their widespread clinical application (de Puig et al., 2017). Current technological innovations, such as employing fluorescent, enzyme-labeled, or surface-enhanced Raman scattering nanoparticles to enhance signal transduction, are significantly improving detection limits (Li J. et al., 2023; Liang et al., 2023) and multiplexing capabilities (Wu et al., 2024). Improvements in LFIA technology primarily focus on signal amplification to enhance sensitivity and detection. These advances enhance the reliability and versatility of LFIA for detecting respiratory pathogens. However, LFIA still faces challenges: (1). Extremely low viral loads during the incubation period for some viruses necessitate integrating complementary techniques to boost sensitivity (Lee et al., 2025); (2). Costs associated with nanomaterial preparation and reading devices may limit widespread adoption (Gong et al., 2023); (3). Complex respiratory secretions, such as those with high viscosity, can clog membrane pores, requiring novel pretreatment methods (Gong et al., 2023).

### 4.3 Molecular biology detection

Over the past two decades, nucleic acid amplification tests (NAATs) have revolutionized the diagnosis of RTIs, achieving unprecedented levels of sensitivity and specificity. These methods have largely replaced traditional techniques as the preferred approach for detecting most viruses and various fastidious bacteria. The following sections will detail the primary nucleic acid amplification testing platforms, ranging from sequencing technologies to emerging innovations, such as clustered regularly interspaced shortpalindromic repeats/CRISPR-associated systems (CRISPR/cas), and Aptamer.

#### 4.3.1 Polymerase chain reaction (PCR) and its derivative technologies

Multiple PCR variant technologies have been developed, significantly enhancing detection sensitivity and throughput through methods such as fluorescence detection, quantitative detection, and multiplex target amplification. Detection limits can as low as 1–10 copies per reaction (Gu et al., 2022). Based on differences in specificity, sensitivity, and applicable scenarios, various PCR techniques are widely employed for the detection of respiratory pathogens. The most clinically prevalent is quantitative PCR (qPCR) technology.

Quantitative PCR serves as the cornerstone of modern molecular diagnostics. By monitoring fluorescence signals during amplification in a closed system, this technique enables the rapid, quantitative detection of specific pathogens within 1–4 h, while minimizing contamination. Its detection limit ranges from 10 to 100 copies/μL (Li J. et al., 2024). One study demonstrated that qPCR achieved a 100% negative predictive value for detecting pathogens in respiratory samples from critically ill pediatric patients (Osborne et al., 2025). Compared to traditional methods, qPCR significantly enhances the detection sensitivity of *S. pneumoniae* in nasopharyngeal swabs or sputum, boosting the positive rate from 0.7% to 10.4% (Almeida et al., 2020). Studies indicate that for patients receiving antibiotic treatment within 72 h prior to admission, qPCR achieves a 78% positive rate for bacterial pathogens, far exceeding the 32% positive rate of culture methods (Gadsby et al., 2016). This demonstrates that empirical antibiotic therapy has a far lesser impact on qPCR detection rates than traditional culture techniques. In diagnosing respiratory fungal infections, qPCR techniques developed from fungal genomic sequences offer advantages such as speed, high sensitivity, and reproducibility. However, primer design requires customization for specific pathogens, potentially overlooking unknown fungal species (Brackin et al., 2021). Due to its high diagnostic sensitivity and specificity, qPCR has become the “gold standard” for respiratory pathogen detection (Chen L. et al., 2021). This technology significantly shortens detection time, supporting early clinical diagnosis and timely intervention. Its primary limitations include insufficient sensitivity for low-copy-number samples and susceptibility to interference from background signals or PCR inhibitors within the reaction system. Consequently, it has become the de facto gold standard for routine pathogen detection, though its reliance on predefined primers limits its ability to identify novel pathogens (Chen L. et al., 2021).

Digital PCR (dPCR) represents an advanced iteration of PCR technology. By partitioning samples into thousands of independent reactions, this method enables absolute nucleic acid quantification without requiring a standard curve and exhibits greater tolerance to PCR inhibitors. This makes dPCR particularly effective for detecting low-abundance pathogens in complex matrices such as sputum. In detecting lower respiratory tract infections in children, dPCR demonstrated a 2.3% higher detection rate than qPCR (Chen X. et al., 2024). Furthermore, dPCR can detect pathogens at low concentrations, exhibiting significantly superior accuracy to qPCR in samples with low concentrations, thereby reducing the risk of false negatives (Yi et al., 2021). In studies of hospital-acquired pneumonia, dPCR enabled early diagnosis and facilitated monitoring of treatment (Merino et al., 2022). However, as a third-generation PCR technology, digital PCR still has limitations, including higher costs and lower throughput, which restrict its application to specialized fields.

#### 4.3.2 Isothermal nucleic acid amplification techniques (INAAT)

Isothermal nucleic acid amplification techniques encompass loop-mediated isothermal amplification (LAMP), recombinase polymerase amplification (RPA), nuclease-assisted circular amplification, and rolling circle amplification (Chen N. et al., 2021). These methods enable nucleic acid amplification at a constant temperature, eliminating the need for a thermal cycler. This characteristic enables rapid, instrument-free testing, making it ideal for POCT and resource-limited settings (Chen N. et al., 2021). The development of multiplex LAMP technology enables simultaneous detection of multiple respiratory pathogens, including SARS-CoV-2 and influenza A/B viruses (Hayashida et al., 2023). The integration of INAAT with the CRISPR-Cas system enables this technology to achieve sub-attomolar sensitivity, marking a major breakthrough in pathogen detection (Zhou et al., 2018). For instance, integrating RPA with the CRISPR/Cas12a system enables visual detection via fluorescence or lateral flow chromatography (Wang et al., 2023c). This integrated approach has demonstrated ultra-high sensitivity requiring only three copies per reaction, with its broad applicability in viral detection extensively validated. The triple-detection system, integrating RPA-CRISPR/Cas12a-fluorescence detection, enabled the rapid identification of RSV (Gong et al., 2022) and HMPV (Qian et al., 2021). These advanced technologies are crucial for early diagnosis of respiratory infections and epidemic control, offering particular advantages during public health emergencies. Challenges persist, however: while certain technologies, such as RPA, have demonstrated sensitivity approaching PCR, their inability to distinguish single-nucleotide differences may compromise pathogen identification accuracy (Tan et al., 2025). Furthermore, current multiplex detection technologies have a limited scope, typically capable of simultaneously detecting only three to four pathogens (Hayashida et al., 2023), making them ill-suited for complex outbreak scenarios. Continuous technological refinement is essential to fully realize their potential in diagnosing respiratory infections and controlling epidemics.

#### 4.3.3 High-throughput sequencing technology

High-throughput sequencing technology, also known as NGS, enables the simultaneous parallel sequencing of large nucleic acid molecules. Applications of NGS technology in infectious disease primarily encompass whole-genome sequencing (WGS), metagenomic sequencing (mNGS), and targeted sequencing (tNGS). WGS technology enables comprehensive genome sequencing of pathogens, precisely identifying their antimicrobial resistance profiles and providing robust evidence for clinical drug decision-making (Allix-Béguec et al., 2018). However, WGS requires the isolation and culture of microorganisms to obtain purified strains. Currently, mNGS and tNGS are the primary sequencing methods for detecting respiratory pathogens.

Metagenomic sequencing, which sequences all nucleic acids in a sample, provides a hypothesis-free detection method. This unbiased characteristic makes it an indispensable tool for diagnosing rare, polymicrobial, or novel infections in immunocompromised or critically ill patients. Takeuchi et al. (2019) employed mNGS technology to detect pathogens missed by conventional methods in bronchoalveolar lavage fluid samples, demonstrating a detection rate of 95.18% and a specificity of 91.30% in such specimens (Diao et al., 2022). The application of NGS technology in infection diagnosis dates back to the 2010s. Initially, mNGS served as a non-targeted approach for viral detection (de Vries et al., 2021), offering unbiased, broad-spectrum pathogen detection capabilities. This technology directly analyzes all genetic material within patient samples, covering multiple pathogen types present in the specimen (Gu et al., 2019). Zinter et al. (2019) successfully identified multiple bacteria, fungi, and viruses in lung samples from immunocompromised children, demonstrating the potential of mNGS in diagnosing complex infections. Leveraging its advantages of evaluating all microorganisms in a single test and delivering rapid results, mNGS has gained widespread adoption in current clinical and research settings (Zhou H. et al., 2021). In fungal detection, mNGS significantly improves positive detection rates compared to traditional methods (Tao et al., 2022), substantially shortens diagnostic cycles, and demonstrates superior detection capabilities for filamentous fungi (e.g., Mucorales, *Aspergillus niger* complex) relative to tNGS technology (Yin et al., 2024). This technology offers distinct advantages in the diagnosis of early-stage infections and among immunocompromised patients, enabling the rapid identification of pathogens associated with severe, refractory, or emerging infectious diseases (Han et al., 2019). Although mNGS can unbiasedly screen all pathogens, its sensitivity for low-abundance pathogens remains limited. Its limitations include high cost, complex bioinformatics analysis, host nucleic acid interference, and challenges in distinguishing colonization from true infection.

Targeted sequencing improves detection sensitivity for specific pathogens. It sequences predefined genomic targets after enrichment with probes. tNGS performs well for low-biomass pathogens, such as *Pneumocystis jirovecii*, which are often missed by conventional sequencing (Yin et al., 2024). In clinical practice, tNGS guides diagnosis and antibiotic therapy for highly pathogenic, rare, or non-colonizing microorganisms, including *Mycobacterium tuberculosis* (*M. tuberculosis*) complex, atypical pathogens, *Aspergillus* species, and non-tuberculous mycobacteria. Treatment regimens changed based on tNGS results for 38.8% of patients. For pathogen detection and antimicrobial resistance testing in severe pneumonia, tNGS results agreed with culture, mNGS, and RT-qPCR (Zhang et al., 2024). Using tNGS highlights its role in evaluating interventions for severe pneumonia and guiding treatment. Its targeted approach reduces cost and turnaround time, making it suitable for detecting pathogens with known resistance markers, such as *M. tuberculosis* complex. In 2023, the World Health Organization added tNGS to its recommended methods for identifying tuberculosis and drug-resistant bacteria (World Health Organization [WHO], 2023b). However, tNGS cannot detect pathogens outside its panel, limiting its usefulness for novel pathogens.

#### 4.3.4 Novel nucleic acid technology

With technological advancements, novel nucleic acid detection methods have emerged, including CRISPR nucleic acid detection technology and in vitro screening techniques inspired by aptamer sensors. These innovations have pioneered new pathways for diagnosing respiratory pathogens.

In 2017, Gootenberg et al. (2017) first reported the development of the CRISPR diagnostic system SHERLOCK (Specific High-Sensitivity Enzymatic Reporter Unlocking) utilizing the trans-cleavage activity of CRISPR/Cas13. This system can detect RNA viruses in samples with varying specificities (Metsky et al., 2020). Unlike traditional methods, this system eliminates the need for Reverse Transcription PCR (RT-PCR) amplification of viral RNA, thereby enhancing detection accuracy. Its core advantage lies in mitigating cross-contamination between samples to a certain extent (Kellner et al., 2019).

Severe Acute Respiratory Syndrome Coronavirus 2 has posed a significant threat to global public health in recent years. While nucleic acid testing has become the gold standard for diagnosing COVID-19 infection, the process is complex, costly, and time-consuming, requiring laboratories and personnel to possess high levels of technical expertise (Xiong et al., 2021). Consequently, rapid diagnostic technologies have emerged to enable early virus screening and containment of viral transmission (Carter et al., 2020). Feng Zhang et al. (2020) demonstrated the practicality of the CRISPR/Cas system for COVID-19 diagnosis, suggesting it could replace traditional PCR methods. Kostyusheva et al. (2022) further elaborated on the system’s potential in infectious disease diagnosis, emphasizing its rapid and low-cost advantages.

Aptamer sensors have emerged as a key research direction due to their high specificity (Chen Z. et al., 2024). Nucleic acid aptamers are single-stranded DNA, RNA fragments, or other nucleic acid analogues capable of specifically binding to target molecules. Functioning as “chemical antibodies,” they exhibit high specificity and affinity, enabling precise recognition of diverse target molecules and demonstrating unique application potential in pathogen detection (Kohlberger and Gadermaier, 2022). Aptamers obtained via Systematic Evolution of Ligands by Exponential Enrichment can be integrated with techniques such as fluorescence resonance energy transfer (Kim et al., 2019), RT-PCR (Liu et al., 2020), surface plasmon resonance (Jia et al., 2018), and capillary electrophoresis (Hu Y. et al., 2025) to evaluate their binding performance with target molecules. Nucleic acid aptamers interact with target molecules through unique structural and conformational changes coupled with signal transduction mechanisms. They can bind smaller target molecules or regions unrecognized by antibodies, offering significant advantages over traditional monoclonal antibodies with targeting and modification capabilities (Wang T. et al., 2019). Aptamers have been developed to detect various respiratory viruses, including influenza, SARS-CoV-2, Ebola, and SARS-CoV-2 (Kim et al., 2020; Kulabhusan et al., 2023; Martin et al., 2024).

## 5 Discussion

The spread of respiratory pathogens has long posed a significant threat to global public health. The prevalent pathogens responsible for acute respiratory infections primarily encompass viruses, bacteria, fungi, and atypical pathogens, such as Mp. In recent years, there has been a notable increase in the emergence of novel pathogens, including MERS-CoV, SARS-CoV-2, multidrug-resistant bacteria, and highly pathogenic avian influenza viruses. These pathogens exhibit diverse transmission routes and a wide host range, which places more stringent demands on the rapid identification and precise source tracing of pathogens. Moreover, latent infections, low-load pathogens, and host immune escape mechanisms further complicate the detection process.

The traditional pathogen culture method has notable advantages in detecting respiratory pathogens. It offers high accuracy in drug-sensitivity tests, comes cheaply, and does not require sophisticated equipment. However, it also has several drawbacks. The detection cycle is long, and its sensitivity is low. When the pathogen load is low, multiple factors, including the specimen type, collection method, pre-analysis medication, and transportation process, can impact the culture of microorganisms, potentially resulting in false-negative results.

Additionally, this method cannot detect some non-culturable pathogens since modern technologies such as sequencing and mass spectrometry have identified certain bacterial species previously not considered to cause RTI. This suggests that while traditional pathogen culture methods remain a crucial component of the clinical microbiology workflow, their limitations will become increasingly evident without the support of modern auxiliary methods.

Immunological methods have emerged as a powerful complement to pathogen culture. They are relatively simple to operate and offer rapid detection. The primary immunological methods include antigen detection and antibody detection. After a pathogen infects the body, antigens are produced. They exhibit excellent specificity; a positive antigen test result typically confirms a diagnosis. Antigen testing can be employed for the early diagnosis of diseases, which is of great significance for prompt intervention. However, antigen testing is easily constrained by sampling. For some pathogens, particularly atypical ones, the infection symptoms may present as a non-productive cough and dryness, leading to a low positive rate in antigen testing. Overall, antigen testing has relatively poor sensitivity and is liable to missed diagnosis. Therefore, a negative antigen test result cannot rule out pathogen infection. For negative results, conducting nucleic acid testing for further screening is advisable. The body typically takes about a week to generate antibodies after being infected with a pathogen. Consequently, antibody testing has limited value for early detection, and false-negative results can occur, especially in patients with weakened immune systems. Nevertheless, antibody testing is useful for retrospective diagnosis in the later stages of a disease and for epidemiological investigations of the population. Overall, with the widespread advancement of molecular biology, the significance of antibody testing is gradually diminishing.

Polymerase chain reaction and its derivative technologies have become the mainstream methods for detecting respiratory pathogens due to their advantages of high sensitivity, high specificity, rapid detection speed, and the ability for quantitative detection. However, they also have some drawbacks. They are relatively costly, demand a high level of expertise from personnel, are susceptible to external environmental contamination during the detection process, require strict quality control and operational standards, and are easily restricted by primer design. In the event of a new virus emerging, the primers need to be redesigned.

Metagenomic sequencing offers a broad detection scope and can identify a wide array of known and unknown pathogens, including rare and newly emerging ones. This provides comprehensive insights into RTI. It is especially well-suited for complex infections or challenging cases, where mNGS shows remarkable advantages. However, tNGS for low-abundance pathogens is easy to miss and may experience non-specific amplification. It is also susceptible to false-positive results caused by environmental aerosols, which undermines the reliability of the results. In the case of NGS, the costs associated with equipment and reagents are substantial. Moreover, interference from the host background complicates the interpretation of results. Data analysis is a complex process with a long analysis cycle and demands a high level of expertise from personnel. A comprehensive assessment in conjunction with clinical manifestations and other test results is required for the pathogens detected through NGS to determine whether they are the target pathogens. This presents certain challenges in interpreting the results.

Clustered regularly interspaced short palindromic repeats/associated systems technology has several remarkable advantages: rapidity, high efficiency, strong specificity, and straightforward operation. It involves a mixed reaction of reagents and samples under normal conditions. It exhibits single-molecular-level sensitivity and can precisely identify the target sequences of specific pathogens. Nevertheless, it necessitates a pre-amplification step, and off-target effects are risky. A molecular diagnostic system based on Cas effectors has been established, further augmenting its application potential in molecular diagnosis. Existing research has combined the CRISPR/Cas system with aptamers. Using aptamer-mediated lysis technology, pathogen nucleic acids can be directly released from nasopharyngeal swabs, eliminating the need for traditional extraction steps. This not only shortens the detection time but also enhances the detection sensitivity. Moreover, it can overcome the limitations of non-nucleic acid target detection, improving both sensitivity and specificity. Aptamers have demonstrated the potential to substitute antibodies in detecting respiratory pathogens. However, issues such as stability, standardization, and cost need to be addressed for their clinical application. In the future, through the integration of interdisciplinary technologies such as CRISPR and nanomaterials, aptamers are expected to become the core components of next-generation diagnostic tools.

In conclusion, it is anticipated that in the future, multiple testing methods or groups will be used in combination to strengthen the capacity to identify pathogens in complex respiratory tract samples. The advancement of portable intelligent devices, such as microscopic technology based on mobile-phone imaging and handheld nanopore sequencers, has facilitated on-site and immediate diagnosis. By leveraging artificial intelligence and deep learning, NGS data analysis can be optimized to predict pathogens’ evolutionary trajectories and drug-resistance mutations. The detection of respiratory pathogens will progress towards the “precision, intelligence, and portability” goals, and the existing bottlenecks are expected to be overcome by integrating interdisciplinary technologies. Simultaneously, establishing a global integrated monitoring system and a data-sharing platform will become the core strategy for addressing emerging infectious diseases and biosecurity threats.

## 6 Conclusion

Over the past few decades, the field of RTI has witnessed remarkable progress, marked by significant breakthroughs in the sensitivity and specificity of pathogen detection. Despite these achievements, certain deficiencies and issues require further attention and resolution. Currently, there is no detection method that is both efficient and convenient while also offering high accuracy and specificity. Moreover, no single method or combination of methods can detect all pathogens. It is imperative to strengthen multi-center cooperation and communication to drive the development of respiratory pathogen detection technology jointly. Addressing these challenges through collaborative innovation is paramount to reducing the immense global burden RTI imposes.
